# Supplementary material for: Identification of photoperiod-induced specific miRNAs in the adrenal glands of Sunite sheep (Ovis aries)
Source: Front Vet Sci. 2022 Jul 22;9:888207. doi: 10.3389/fvets.2022.888207 (PMC9354845; doi:10.3389/fvets.2022.888207)
Supplement: Supplementary file 1 [file Data_Sheet_1.ZIP › Supplementary file/miRDeep2 file.zip!/miRDeep2 file/Know_Structure/SP42A2/oar-mir-181a-2.pdf]

miRBase precursor : oar-mir-181a-2
Total read count : 17831
oar-miR-181a read count : 17635
remaining reads : 196

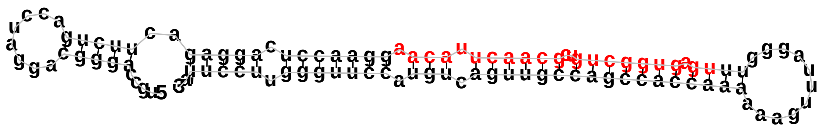

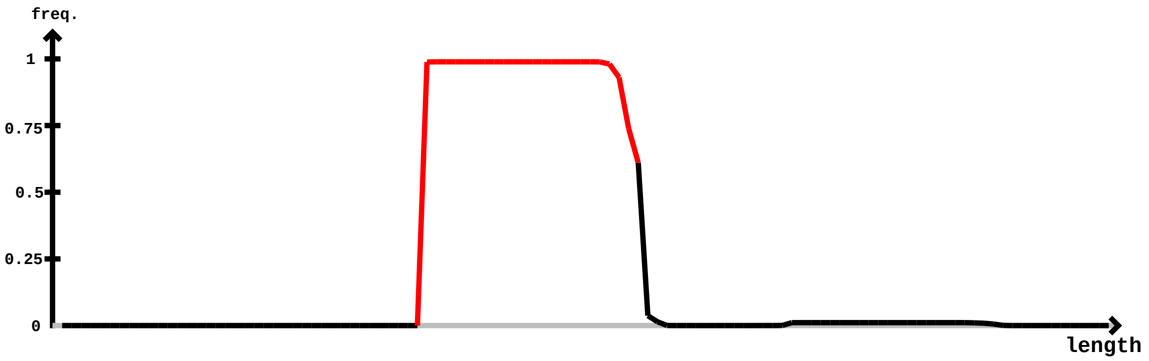


```
                                     oar-miR-181a
5'- ugccagggcaggauccagucuucagaggacuccaaggaacauucaacgcugucggugaguuugggauuugaaaaaaccaccgaccguugacuguaccuuggguuccuua   -3'    exp
    ....(((((........)))))..(((((.(((((((.(((.((((((..(((((((.((((...........))))))))))))))))).))).))))))).))))).   reads      mm         sample
    .....................................aacauucaacgcugucgg......................................................   6          0          seq
    .....................................aacauucaacgcugucggu.....................................................   145        0          seq
    .....................................aacauucaacgcugucggug....................................................   894        0          seq
    .....................................aacauucaacgcugucgguga...................................................   3410       0          seq
    .....................................aacauucaacgcugucggugag..................................................   2294       0          seq
    .....................................aacauucaacgcugucggugagu.................................................   10210      0          seq
    .....................................aacauucaacgcugucggugaguu................................................   409        0          seq
    .....................................aacauucaacgcugucggugaguuu...............................................   250        0          seq
    .....................................aacauucaacgcugucggugaguuug..............................................   3          0          seq
    ......................................acauucaacgcugucgguga...................................................   1          0          seq
    ......................................acauucaacgcugucggugag..................................................   1          0          seq
    ......................................acauucaacgcugucggugagu.................................................   10         0          seq
    ......................................acauucaacgcugucggugaguuu...............................................   1          0          seq
    ........................................auucaacgcugucggugagu.................................................   1          0          seq
    ........................................................................aaaaccaccgaccguugacugu...............   1          0          seq
    ..........................................................................aaccaccgaccguugacugua..............   3          0          seq
    ..........................................................................aaccaccgaccguugacuguac.............   4          0          seq
    ...........................................................................accaccgaccguugacugu...............   15         0          seq
    ...........................................................................accaccgaccguugacugua..............   20         0          seq
    ...........................................................................accaccgaccguugacuguac.............   51         0          seq
    ...........................................................................accaccgaccguugacuguacc............   91         0          seq
    ...........................................................................accaccgaccguugacuguaccu...........   10         0          seq
    ............................................................................ccaccgaccguugacuguacc............   1          0          seq
```
